# Preoperative evaluation of post-hepatectomy liver failure in hepatocellular carcinoma based on gadoxetic acid-enhanced MRI

**DOI:** 10.3389/fonc.2026.1789478

**Published:** 2026-06-29

**Authors:** Qin Wang, Qian Li, Shixue Li, Zheng Li, Xia Yao, Hehan Tang, Yi Wei, Wei Ren, Liping Deng, Yuan Yuan

**Affiliations:** 1 Department of Radiology, West China Hospital, Sichuan University, Chengdu, China; 2 Department of Clinical Application, General Electric Healthcare China Co Ltd, Beijing, China

**Keywords:** gadoxetic acid-enhanced MRI, hepatocellular carcinoma, post-hepatectomy liver failure, prediction model, quantitative assessment of liver function

## Abstract

**Purpose:**

Post-hepatectomy liver failure (PHLF) is a severe complication after hepatic resection. This explored the value of gadoxetic acid-enhanced MRI quantitative imaging biomarkers of liver function for the preoperative prediction of PHLF in HCC patients.

**Methods and Materials:**

This retrospective study included 107 patients undergoing hepatectomy for HCC. All had gadoxetic acid-enhanced MRI preoperatively. Quantitative imaging biomarkers of liver function, including relative liver enhancement (RLE), hepatic uptake index (HUI), contrast uptake index (CUI) and liver-to-spleen index (LSI) were calculated. Intraclass correlation coefficient (ICC) was applied to verify the reproducibility of ROI delineation. Principal Component Analysis (PCA) was used to handle multicollinearity among imaging biomarkers. Multivariate binary logistic regression was used to identify independent risk factors for PHLF and establish preoperative prediction models. Receiver operating characteristic (ROC) curve analysis with DeLong test was performed to compare the predictive efficacy of different models, and decision curve analysis (DCA) was adopted to evaluate the clinical net benefit of the prediction models.

**Results:**

Among the 107 patients (95 males, 12 females; mean age 56.2 ± 12.4 years), 29 were PHLF positive, and 78 were negative. Stratified multivariate binary logistic regression analysis revealed that RLE, CUI, HUI and LSI were all independent predictors of PHLF when respectively incorporated into independent models. (RLE OR< 0.001, 95% CI< 0.001-0.368, *p* = 0.028; CUI OR 0.028, 95% CI 0.005-0.149, *p<* 0.001; HUI OR 0.998, 95% CI 0.996-0.999, *p<* 0.001; LSI OR 0.033, 95% CI 0.007-0.158, *p<* 0.001). Factor analysis was used to integrate RLE, CUI, HUI, and LSI into a comprehensive index (RLE−CUI−HUI−LSI), which also demonstrated independent predictive value for PHLF. (RLE−CUI−HUI−LSI OR<0.001, 95% CI< 0.001-0.010, *p* < 0.001) Among the models, the RLE-TBil model (AUC 0.997) showed the strongest predictive ability for PHLF. All quantitative index models significantly outperforming conventional liver function indices. DeLong test for pairwise comparisons and DCA confirmed the superior predictive efficacy and clinical net benefit of these quantitative models.

**Conclusion:**

Quantitative imaging biomarkers of liver function based on gadoxetic acid-enhanced MRI represents a vital preoperative evaluation method.

## Introduction

Hepatocellular carcinoma (HCC) is a primary malignant liver cancer with a mortality rate of up to 8.3%, ranking third among all global malignancies (1–3). Currently, hepatectomy is considered the primary curative treatment for HCC (4). However, post-hepatectomy liver failure (PHLF) remains the leading cause of postoperative mortality, accounting for 41-62% of postoperative deaths (5, 6). Accordingly, accurate preoperative risk evaluation of PHLF is crucial to prevent life-threatening complications after hepatectomy (3, 7).

The occurrence of PHLF is closely associated with the volume of the future liver remnant (FLR) and the degree of hepatic impairment. In general, the assessment of hepatic reserve function is primarily based on biochemical parameters such as the model for end-stage liver disease (MELD) score, albumin-bilirubin index (ALBI) score, and liver fibrosis-related indices. However, these models do not account for hepatic volume (8–11). FLR volume assessment based on computed tomography (CT) or magnetic resonance imaging (MRI) is effective; however, it cannot accurately predict PHLF in patients with hepatic steatosis or fibrosis (12). Hence, simultaneous assessment of FLR and hepatic reserve function is essential for reliable PHLF risk evaluation.

Gadoxetic acid-enhanced MRI is also widely used for the preoperative assessment of liver function (12). Unlike conventional imaging examinations, gadoxetic acid, as a paramagnetic hepatobiliary-specific contrast agent, enables simultaneous morphological and functional evaluation of the liver based on hepatocyte transporter expression (13). Multiple studies have demonstrated its significant clinical value in predicting PHLF (14). Previous studies have shown a strong correlation between quantitative hepatobiliary phase (HBP) measurements and established liver function parameters. Four quantitative imaging biomarkers of liver function—relative liver enhancement (RLE), hepatic uptake index (HUI), contrast uptake index (CUI), and liver-to-spleen index (LSI) —have been proven to correlate with liver function (15, 16). To date, some studies have verified the predictive ability of RLE and HUI for PHLF. However, to our knowledge, no study has explored the predictive ability of CUI and LSI for PHLF, nor has any study systematically compared the performance of these four quantitative imaging biomarkers for liver function (17, 18).

The aim of this study is to explore the predictive ability of four quantitative imaging biomarkers of liver function (RLE, CUI, HUI, LSI) based on gadoxetic acid-enhanced MRI in PHLF evaluation (8).

## Patients and methods

### Patients

This retrospective study was conducted in accordance with the ethical guidelines of the 1975 Declaration of Helsinki. Ethical approval was granted by the Institutional Review Board of West China Hospital, Sichuan University. The requirement for written informed consent was waived due to the retrospective nature of the study.

**Figure 1:**
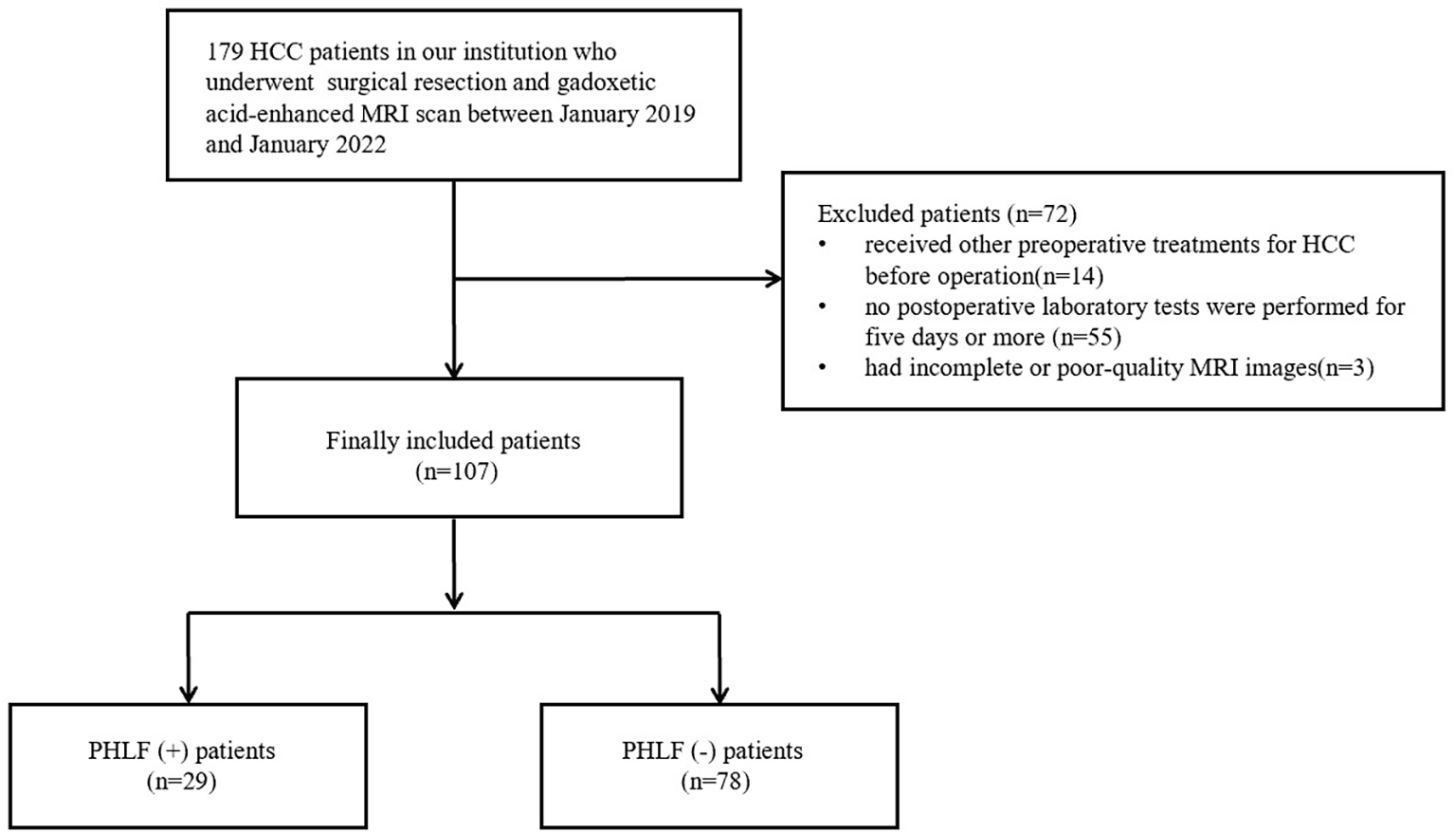
The Flowchart for the study population.

Patients who underwent hepatectomy for HCC between January 2019 and January 2022 at West China Hospital of Sichuan University were considered for inclusion in this study. The inclusion criteria were as follows: (1) hepatectomy as the initial treatment for primary liver lesions, which were defined as initial lesions with no evidence of extrahepatic primary tumors on imaging, excluding recurrent liver tumors.; (2) the diagnosis of HCC was confirmed by postoperative pathology; (3) the time interval between preoperative gadoxetic acid-enhanced MRI and surgery was less than 4 weeks; (4) biochemical liver function parameters (1 + 4) and routine coagulation laboratory tests were performed within 1 week before and 5 days after surgery. Patients were excluded if they (1) received other preoperative treatments for HCC before operation(n=14), (2) had no postoperative laboratory tests performed for 5 days or more (n=55), or (3) had incomplete or poor-quality MRI images (plain T1WI or HBP sequence images were either missing or exhibited excessive respiratory motion artifacts, making anatomical structures unrecognizable.) (n=3). Finally, 107 patients were enrolled in this study (**Figure 1**).

### Methods

#### MRI examination

All patients underwent gadoxetic acid-enhanced MRI using a 3.0T MR scanner (MR 750, GE Healthcare) with an 18-channel phased-torso array coil. In this study, all patients were instructed to fast for 6–8 h before the MR examination and were placed in a supine position with the scan covering the entire liver and spleen.

A three-dimensional fat-suppressed T1-weighted gradient echo sequence (LAVA) was used, with the following parameters: TR = 6.1 ms, TE = 2.7 ms, flip angle = 15°, slice thickness = 3.0 mm, matrix size = 300×256, field of view = 380×320, and number of excitations = 1.0. Axial dynamic images were acquired before and during the late arterial(30–35 s), portal venous (50–60 s), transitional (2–5 min), and hepatobiliary (20 min) phases after contrast administration (19). The contrast agent was a hepatobiliary-specific contrast agent gadoxetic acid disodium (Gd-EOB-DTPA) (Primovist^®^; Bayer Healthcare), which was administered intravenously at a dose of 0.025 mmol/kg with a rate of 1–2 mL/s, followed immediately by a 20 mL flush of 0.9% saline to clear the contrast agent from the venous catheter (20). The arterial phase imaging time was determined using an automated contrast material bolus tracking technique (7 s after the arrival of the contrast bolus in the celiac trunk) or multiple arterial phase imaging technique (acquired during an 18-s breath hold with a 20-s delay after contrast injection and reconstructed with a temporal resolution of 3 s).

#### PHLF criteria

According to the International Study Group for Liver Surgery (ISGLS), PHLF is defined as impairment of the liver to maintain synthesis, excretion and detoxification. The diagnostic criteria are a high international normalized ratio (INR) on or after 5 days post-hepatectomy, accompanied by hyperbilirubinemia (9). In patients with preoperative liver function abnormalities, baseline values were used as references for postoperative evaluation. In this study, according to the laboratory criteria of West China Hospital of Sichuan University (INR >1.15 and serum total bilirubin (TBil) >28.0 μmol/L), 29 patients were classified as PHLF-positive and 78 as PHLF-negative.

#### Imaging analysis

##### Quantitative imaging biomarkers of liver function

Two board-certified abdominal radiologists (with 10 and 12 years of experience in abdominal imaging, respectively), who were blinded to all clinical, pathological, and follow-up results, independently interpreted the gadoxetic acid-enhanced MR images.

The regions of interest(ROIs): Quantitative measurements were performed on unenhanced and HBP images for each patient. ROIs were drawn on the maximum plane of the remaining liver after hepatic resection according to the surgical resection method and in the homogeneous parenchymal regions in three contiguous planes (superior and inferior to this plane). The ROI was 4–5 mm from the edge of the liver, excluding the edge, with strict exclusion of all macroscopically identifiable large vessels (main trunks and primary branches of the portal vein, hepatic artery, and hepatic veins), dilated intrahepatic bile ducts, and focal parenchymal lesions. The mean value of the three liver ROIs was calculated (15). Concurrently, the ROIs of the spleen and paraspinal muscle at the same anatomical level were delineated, with an ROI size of 2–5 mm^2^.

Measurement of liver volume: Liver volume measurements were performed on HBP images of patients using the volume analysis tool on a GE AW workstation.

Quantitative imaging biomarkers of liver function were calculated using the following formulas (13, 21):


$$\text{RLE}=(SI_{Liver\;enh\;HBP}-SI_{Liver\;unenh\;})/SI_{Liver\;unenh}\times 100$$

$$\text{CUI}=(SIR_{enh\;}/SIR_{unenh\;})\text{; SIR}=(SI_{Liver\;}/SI_{paraspinal\;muscle\;})$$

$$\text{HUI}=Volume_{Liver\;}[\frac{SI_{Liver\;enh\;HBP}}{SI_{Spleen\;enh\;HBP}}-1]$$

$$\text{LSI}=SI_{Liver\;enh\;HBP}/SI_{Spleen\;enh\;HBP}$$

*RLE* relative liver enhancement, *CUI* contrast uptake index, *HUI* hepatic uptake index, *LSI* liver-to-spleen index, *SI* signal intensity, *SIR* SI ratio, is calculated as a ratio of the hepatic SI to the paraspinal muscle SI separately on unenhanced images and the ratio of the hepatic SI to the paraspinal muscle SIR on hepatocyte-phase images.

##### The FLR volume

Preoperative gadoxetic acid-enhanced MRI HBP images were imported into 3D Slicer software. The segmentation method was used to simulate the surgical resection plan based on upper abdominal CT examination performed within 2 weeks before surgery, to obtain the FLR volume after resection. The reconstructed volume model excluded the gallbladder, inferior vena cava, and major branches of intrahepatic vascular structures, but included biliary tract structures.

#### Calculation of MELD model and ALBI score

MELD score was calculated on INR, serum creatinine (Cre), and TBil, calculated with the formula (22–24):

MELD score = 9.57 ×log_e_ (Cre, mg/dL)+3.78 ×log_e_ (TBil, mg/dL)+11.20 ×log_e_ (INR)+6.43×(etiology:0 if cholestatic or alcoholic, 1 otherwise).

ALBI score was calculated on TBil and albumin, calculated with the formula (25, 26):

ALBI score =0.66×log_10_(TBil, μmol/L)-0.085(albumin, g/L).

#### Statistical analysis

Continuous variables were expressed as means ± standard deviations or medians (interquartile ranges) and compared using the independent-samples t-test or the Mann-Whitney U test. The intraclass correlation coefficient (ICC) was used to assess the inter- and intraobserver agreements of key quantitative imaging biomarkers. First, candidate variables associated with PHLF were screened using univariate logistic regression analysis. In the univariate logistic regression, Cook’s distance and studentized residuals were calculated to identify influential observations and outliers. A Cook’s distance > 1 or a studentized residual > 3 was considered indicative of a strong influential case. A sensitivity analysis was performed by excluding the identified outlier in order to verify the stability of the model calibration. The linearity assumption for continuous variables in the logistic regression was tested using the Box-Tidwell method. If the interaction term of the variable and its natural logarithm was statistically significant (p< 0.05), thus indicating a violation of linearity, a quadratic term was introduced for the sensitivity analysis to account for potential non-linear relationships. Multicollinearity among the candidate variables was assessed using the variance inflation factor (VIF), with a VIF > 10 indicating significant multicollinearity. To address multicollinearity and reduce dimensionality, a single comprehensive factor was extracted from highly correlated liver function imaging variables using factor analysis. Subsequently, multivariate logistic regression analysis was performed to identify the independent risk factors for PHLF and construct predictive models. The predictive performance of each model was evaluated using receiver operating characteristic (ROC) curve analysis, by calculating the area under the ROC curve (AUC), optimal cutoff value, sensitivity, specificity, positive predictive value (PPV), and negative predictive value (NPV). DeLong’s test was used to compare the differences in AUCs between the models. Internal validation was performed using 1000 bootstrap resamples to evaluate model stability and overfitting. Model calibration was assessed using the Hosmer–Lemeshow (H-L) test. The clinical utility was further evaluated using decision curve analysis (DCA), which quantified the net clinical benefit of each model across a range of threshold probabilities. Stratified analysis, calibration curve visualization, precision-recall curve analysis, and misclassification analysis of true-positive and false-positive cases were applied only to the final selected predictive model to assess its robustness and clinical interpretability. All statistical analyses were performed using SPSS 25.0 (IBM Corp., Armonk, NY, USA) and R 4.5.3 software. A two−sided P-value< 0.05 was considered statistically significant.

## Results

### Patient characteristics and group differences

**Figure 2:**
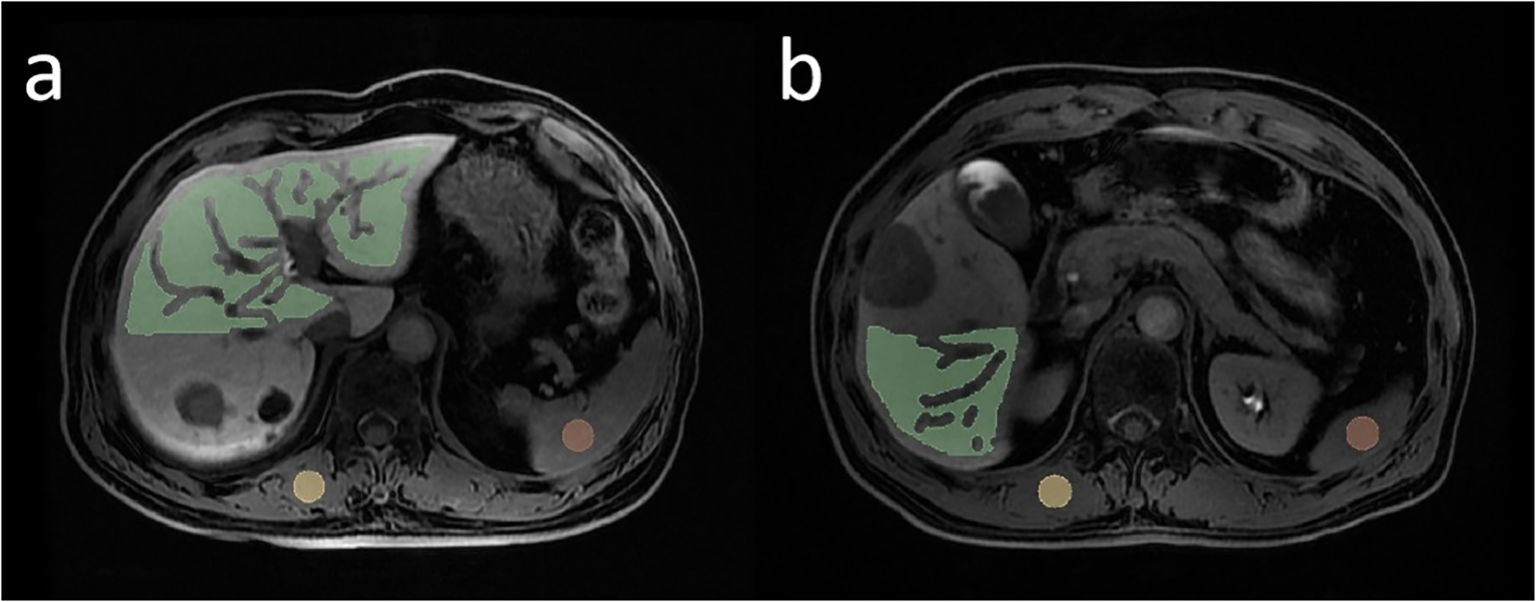
MRI images of HBP phase in two HCC patients. Hepatic regions of ROIs (green) were manually drawn on the maximum cross-sectional plane of the remnant liver after hepatectomy, as well as on two adjacent planes centered on this reference plane. All hepatic ROIs were placed 4–5 mm away from the liver edge to exclude marginal tissue, with strict avoidance of visually identifiable main trunks and primary branches of large hepatic vessels, dilated bile ducts, tubular structures, and focal lesions. Splenic (red) and paraspinal muscle (yellow) reference ROIs were outlined at the corresponding anatomical levels, with a standardized size of 2–5 mm² for all reference ROIs. Quantitative parameters including RLE, CUI, HUI, and LSI were calculated based on the averaged values of all hepatic ROIs. **(a)** A 66-year-old HCC patient without PHLF, with RLE = 4.90, CUI = 5.84, HUI = 4005.42, and LSI = 3.39. **(b)** A 50-year-old HCC patient with PHLF, with RLE = 0.35, CUI = 0.55, HUI = 1089.94, and LSI = 0.81.

In this study, 107 patients who underwent hepatic cancer resection were enrolled and divided into two groups based on the occurrence of PHLF (**Figure 1**). The PHLF (+) group comprised 29 patients (mean age: 55.59 ± 12.58 years), and the PHLF (-) group comprised 78 patients (mean age: 56.46 ± 12.44 years). Significant intergroup differences were observed in RLE (*p* < 0.001), CUI (*p* < 0.001), HUI (*p* < 0.001), LSI (*p* = 0.003), platelet count (PLT) (*p* = 0.023), ALBI score (*p* = 0.028), MELD score (*p* = 0.034), and TBil (*p* < 0.001) (**Table 1**). **Figure 2** shows the typical imaging features of the patients in the PHLF (+) and PHLF (-) groups.

**Table 1:** Clinical characteristics and quantitative indicators of liver function.

Variables	PHLF(-)(n=78)	PHLF(+)(n=29)	*p* value
Age(years)	56.46 ± 12.44	55.59 ± 12.58	0.693
Gender	1.00(1.00,1.00)	1.00(1.00,1.00)	0.390
RLE	2.61(2.12,3.82)	0.40(0.18,0.55)	<0.001^*^
CUI	2.64(2.16,3.39)	1.04(0.84,1.30)	<0.001^*^
HUI	2338.58 ± 1792.11	535.36 ± 637.66	0.001^*^
LSI	2.71 ± 1.16	1.41 ± 0.45	0.003^*^
FLR (cm³)	976.79 ± 241.68	832.89 ± 274.10	0.166
PLT(×10^9^/L)	144.06 ± 84.08	133.24 ± 55.40	0.023^*^
ALBI score	-2.92(-3.18, -2.68)	-2.73(-2.96, -2.39)	0.028^*^
MELD score	3.99(2.52,6.35)	5.40(4.15,7.10)	0.034^*^
INR	1.03 ± 0.09	1.05 ± 0.98	0.629
PT(s)	11.40(10.90,12.00)	11.50(10.85,12.30)	0.656
TBil (μ mol/L)	12.45(9.38,16.43)	19.30(12.55,24.15)	<0.001^*^
ALT(IU/L)	30.00(20.75,42.00)	33.00(22.00-50.00)	0.250
AST(IU/L)	28.00(22.00,38.00)	31.00(23.50-61.50)	0.058
Hepatectomy Extent	0.00(0.00,1.00)	0.00(0.00,1.00)	0.729

Data are represented in mean ± SD or frequency (%). And Data were evaluated by independent samples t-test or the Mann-Whitney U test for continuous variables. ^*^Referred to *p*<0.05.

*RLE,* relative liver enhancement; *CUI,* contrast uptake index; *HUI,* hepatic uptake index; *LSI,* liver-to-spleen index; *FLR,* future liver remnant; *PLT,* platelet count; *ALBI,* albumin-bilirubin; *MELD,* model for end-stage liver disease; *INR,* international normalized ratio; *PT,* prothrombin time; *TBil,* total bilirubin; *ALT,* alanine aminotransferase; *AST* aspartate aminotransferase.

To assess the reliability of the radiological quantitative indices, ICC analysis was used to evaluate the interobserver consistency of the RLE, CUI, HUI, and LSI measurements. The results indicated excellent interobserver agreement for all four indices, with all ICC values exceeding 0.90 (all *p* < 0.001), confirming the high reproducibility and stability of the extracted imaging parameters.

Additionally, severe multicollinearity was identified between albumin and ALBI score, as evidenced by a high VIF (91.231). Albumin was excluded from the multivariate model construction to avoid potential bias in subsequent regression analyses.

### Univariate and multivariate analysis of PHLF predictors

Univariate logistic regression analysis was performed to identify the potential predictors of PHLF. The results showed that PHLF was significantly associated with RLE (Odds Ratio (OR) 0.010; 95% confidence interval (CI) 0.001-0.091; *p* < 0.001), CUI (OR 0.023; 95% CI 0.005-0.110; *p* < 0.001), HUI (OR 0.998; 95% CI 0.997-0.999; *p* < 0.001), LSI (OR 0.058; 95% CI 0.018-0.191; *p* < 0.001), TBil (OR 1.142;95% CI 1.059-1.231; *p* = 0.001), ALBI score (OR 4.130; 95% CI 1.468-11.616; *p* = 0.007), and MELD score (OR 1.167; 95% CI 1.006-1.354; *p* = 0.042). The H-L test was further performed to evaluate the goodness-of-fit of each univariate model. The results showed that, except for the CUI model which exhibited suboptimal goodness-of-fit (*p* < 0.001), the HL test *p* values of all other models were greater than 0.05, indicating satisfactory goodness-of-fit of the univariate predictive models for RLE, HUI, LSI, TBil, ALBI, and MELD. To explore the cause of the suboptimal fit in the CUI model, we performed residual and influential case diagnostics using Cook’s distance and studentized residuals. One extremely influential outlier (Cook’s distance = 1.47, studentized residual = 3.35) was identified. After excluding this single case, the H-L test p-value of the CUI model improved to 0.389, confirming that poor calibration was driven solely by this outlier rather than by the fundamental lack of predictive value of CUI. Notably, the CUI remained significantly associated with PHLF (*p* < 0.001) after excluding this outlier. Additionally, the Box-Tidwell test for the linearity assumption showed a significant interaction term (*p* = 0.002), indicating a non-linear relationship between CUI and PHLF. This non-linearity was successfully accounted for in the sensitivity analysis by including a quadratic term of the CUI, which remained significant (*p* = 0.004) without altering the direction or significance of the primary association. Based on these findings, the CUI variable demonstrated a stable independent predictive value despite the initial poor fit, and was therefore retained in the comprehensive predictive model. In addition, PLT (OR 0.998; 95% CI 0.992-1.004; *p* = 0.519) was not significantly associated with PHLF in univariate analysis (**Table 2**). Given the severe multicollinearity among the quantitative imaging variables RLE, CUI, HUI, and LSI (VIF > 10), two analytical strategies were adopted for multivariate logistic regression analysis to ensure the robustness of the results: stratified subgroup analysis and an integrated factor analysis-based combined model.

**Table 2:** Univariate analysis results for PHL predictors.

Variables	Univariate analysis		H-L test *p* value
	OR (95% CI)	p value	
RLE	0.010(0.001-0.091)	<0.001^*^	0.106
CUI	0.023(0.005-0.110)	<0.001^*^	<0.001
HUI	0.998(0.997-0.999)	<0.001^*^	0.650
LSI	0.058(0.018-0.191)	<0.001^*^	0.445
PLT (10^×9^/L)	0.998(0.992-1.004)	0.519	0.372
TBil (μ mol/L)	1.142(1.059-1.231)	0.001^*^	0.152
ALBI score	4.130(1.468-11.616)	0.007^*^	0.457
MELD score	1.167(1.006-1.354)	0.042^*^	0.070

^*^ Referred to *p*<0.05; *OR,* odds ratio; *CI* confidence interval, *H-L* hosmer–lemeshow, *RLE,* relative liver enhancement; *CUI,* contrast uptake index; *HUI,* hepatic uptake index; *LSI,* liver-to-spleen index; *PLT,* platelet count; *TBil,* total bilirubin; *ALBI,* albumin-bilirubin; *MELD,* model for end-stage liver disease.

### Stratified subgroup analysis

To eliminate collinearity interference, RLE, CUI, HUI, and LSI were separately incorporated into four independent multivariate logistic regression models, with TBil, ALBI score, and MELD score included as covariates in each model: RLE group: RLE (OR<0.001, 95% CI< 0.001-0.368, *p* = 0.028) and TBil (OR 2.553, 95% CI 1.032-6.315, *p* = 0.043) were identified as independent predictors of PHLF, whereas ALBI score and MELD score showed no independent predictive value; CUI group: Only CUI (OR 0.028, 95% CI 0.005-0.149, *p* < 0.001) was an independent predictor of PHLF, with no independent association observed for TBil, ALBI score, or MELD score; HUI group: HUI (OR 0.998, 95% CI 0.996-0.999, *p* < 0.001) and TBil (OR 1.190, 95% CI 1.031-1.372, *p* = 0.017) were confirmed as independent predictors of PHLF, with no significant independent effects for ALBI score or MELD score; and LSI group: LSI (OR 0.033, 95% CI 0.007-0.158, *p* < 0.001) and TBil (OR 1.166, 95% CI 1.009-1.348, *p* = 0.037) were independent predictors of PHLF, while ALBI score and MELD score had no independent predictive significance (**Table 3**).

**Table 3:** Multivariate analysis results of predictors in four groups.

Groups	Variables	Multivariate analysis		H-L test *p* value
		OR (95% CI)	*p* value	
RLE group	RLE	<0.001(<0.001-0.368)	0.028^*^	1.000
	TBil (μ mol/L)	2.553(1.032-6.315)	0.043^*^	
	ALBI score	0.088(0.001-14.823)	0.352	
	MELD score	0.655(0.281-1.527)	0.327	
CUI group	CUI	0.028(0.005-0.149)	<0.001^*^	0.729
	TBil (μ mol/L)	1.153(0.967-1.374)	0.112	
	ALBI score	2.587(0.430-15.554)	0.299	
	MELD score	0.954(0.697-1.306)	0.769	
HUI group	HUI	0.998(0.996-0.999)	<0.001^*^	0.873
	TBil (μ mol/L)	1.190(1.031-1.372)	0.017^*^	
	ALBI score	5.409(0.803-36.420)	0.083	
	MELD score	0.965(0.736-1.266)	0.799	
LSI group	LSI	0.033(0.007-0.158)	<0.001^*^	0.627
	TBil (μ mol/L)	1.166(1.009-1.348)	0.037^*^	
	ALBI score	7.043(0.888-55.863)	0.065	
	MELD score	0.986(0.748-1.301)	0.922	
RLE-CUI-HUI-LSI group	RLE-CUI-HUI-LSI	<0.001(<0.001-0.010)	<0.001*	0.817
	TBil (μ mol/L)	1.213(0.978-1.503)	0.079	
	ALBI score	29.211(0.621-1373.249)	0.086	
	MELD score	0.918(0.593-1.422)	0.701	

^*^ Referred to *p*<0.05; *OR*, odds ratio; *CI*, confidence interval; *H-L*, hosmer–lemeshow; *RLE*, relative liver enhancement; *CUI*, contrast uptake index; *HUI*, hepatic uptake index; *LSI*, liver-to-spleen index; *TBil*, total bilirubin; *ALBI*, albumin-bilirubin; *MELD*, model for end-stage liver disease.

### Principal component analysis to derive a composite imaging score

To further address multicollinearity and synthesize the predictive information of the four quantitative imaging variables, a comprehensive combined index (RLE-CUI-HUI-LSI) was derived from RLE, CUI, HUI, and LSI using principal component analysis (PCA). The Kaiser-Meyer-Olkin (KMO) value was 0.724, and Bartlett’s test of sphericity was significant (*p* < 0.001), confirming the suitability of the data for factor analysis. The first principal component (PC1) was extracted as the composite imaging score and explained 83.634% of the total variance. The factor loadings for the RLE, CUI, HUI, and LSI were 0.863, 0.930, 0.937, and 0.926. This combined index was then included in the multivariate logistic regression model with TBil, ALBI score, and MELD score as covariates (VIF = 1.081–1.735; no obvious multicollinearity). The results showed that the RLE-CUI-HUI-LSI combined index (OR< 0.001, 95% CI< 0.001-0.010, *p* < 0.001) was an independent predictor of PHLF, whereas TBil, ALBI score, and MELD score had no independent predictive value in this integrated model (**Table 3**).

### Performance, validation and clinical utility of predictive models

**Figure 3:**
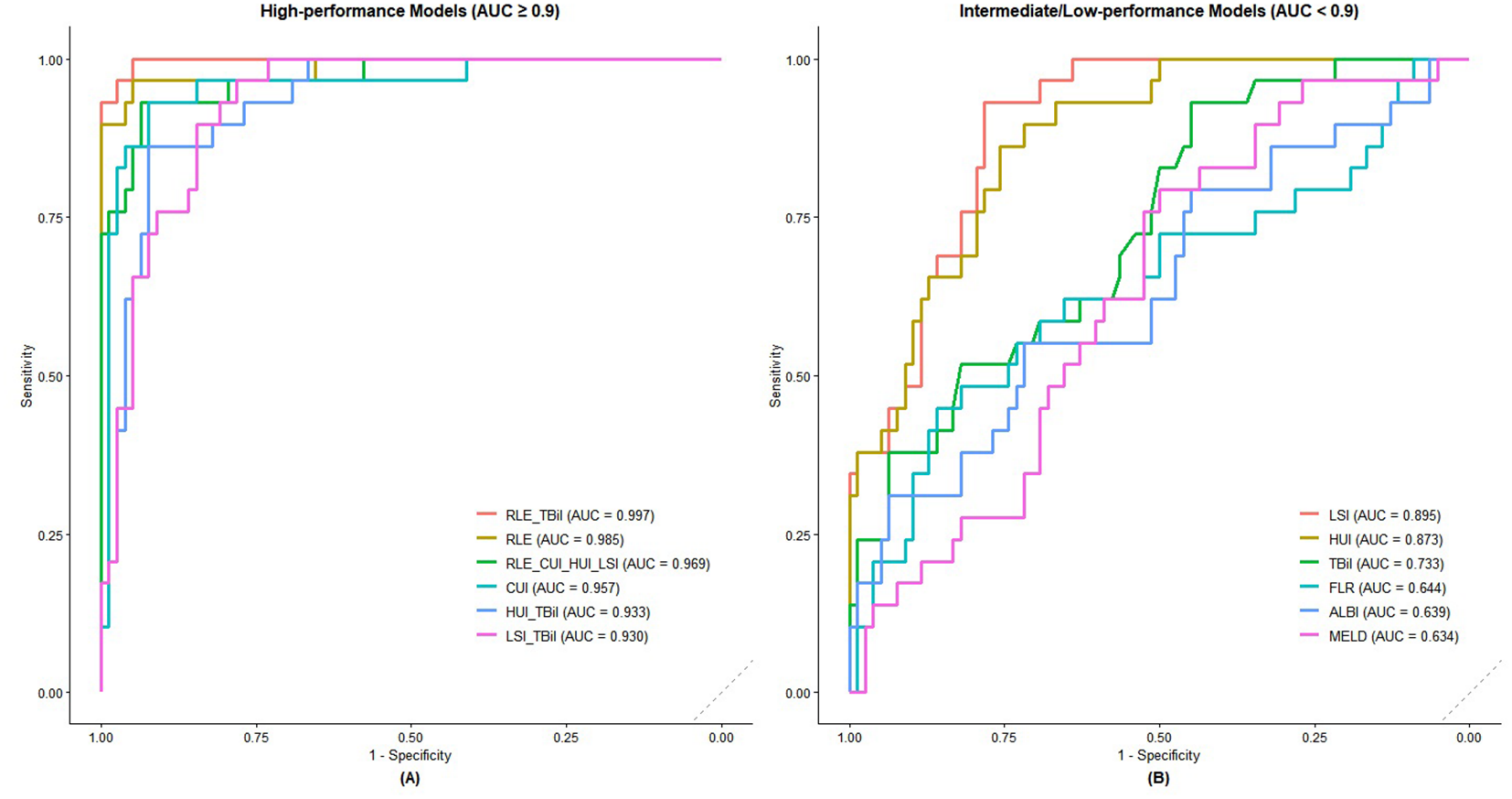
ROC curves of 12 models for predicting PHLF. **(A)** High-performance models (AUC ≥ 0.9), including RLE-TBil, RLE, RLE-CUI-HUI-LSI, CUI, HUI-TBil, and LSI-TBil. **(B)** Intermediate/low-performance models (AUC< 0.9), including LSI, HUI, TBil, FLR, ALBI, and MELD. The x-axis represents 1 - specificity, and the y-axis represents sensitivity. The curves show the discriminative performance of 12 prediction models, with AUC values labeled in the legend. The RLE-TBil model (AUC 0.997) demonstrated the highest predictive accuracy for PHLF.

Based on the results of multivariate logistic regression analysis, several predictive models for PHLF were constructed, including four single quantitative imaging biomarker models of liver function, three combined models, and one comprehensive imaging biomarker model derived from factor analysis (RLE-CUI-HUI-LSI). Three conventional liver function indices were also included for comparison. The predictive performances of all models were evaluated using ROC curve analysis, and the ROC curves are shown in **Figure 3**. In order to improve the readability and avoid visual clutter, the 12 models were stratified into two groups based on their AUC values for presentation: high-performance models (AUC ≥ 0.9) and intermediate/low-performance models (AUC< 0.9). ROC curves of the high-performance group (6 models, including RLE-TBil, RLE, RLE-CUI-HUI-LSI, CUI, HUI-TBil, and LSI-TBil) are shown in **Figure 3A**; curves of the intermediate/low-performance group (6 models, including LSI, HUI, TBil, FLR, ALBI, and MELD) are shown in **Figure 3B**. All models based on quantitative imaging biomarkers of liver function exhibited favorable predictive performance, which was significantly superior to that of conventional liver function indices. The RLE-TBil combined model achieved the optimal predictive performance, with an AUC of 0.997 (95% CI 0.993–1.000), sensitivity of 100%, specificity of 94.9%, a positive predictive value (PPV) of 0.8794, and a negative predictive value (NPV) of 1.0000. The remaining imaging-based models, including RLE, RLE-CUI-HUI-LSI, CUI, HUI-TBil, and LSI-TBil, also showed high predictive efficacy, whereas conventional liver function indices, including TBil, ALBI score, MELD score, and FLR showed markedly lower AUC values with poor predictive efficacy (**Table 4**).

**Table 4:** Diagnostic performance of individual predictive models for PHLF.

Variables	AUC (95% CI)	Cutoff	Sensitivity (%)	Specificity (%)	PPV	NPV	H-L test *p* value
RLE	0.985(0.961-1.000)	1.334	96.6%	94.9%	0.8757	0.9869	0.106
CUI	0.957(0.911-1.000)	1.635	93.1%	92.3%	0.8180	0.9730	<0.001
HUI	0.873(0.806-0.940)	1205.269	86.2%	75.6%	0.5677	0.9364	0.650
LSI	0.895(0.837-0.952)	2.003	93.1%	78.2%	0.6136	0.9682	0.445
TBil (μ mol/L)	0.733(0.630-0.837)	11.350	93.1%	44.9%	0.3858	0.9460	0.152
RLE-TBil	0.997(0.993-1.000)	0.086	100%	94.9%	0.8794	1.0000	1.000
HUI-TBil	0.933(0.886-0.980)	0.459	86.2%	92.3%	0.8063	0.9473	0.173
LSI-TBil	0.930(0.884-0.976)	0.141	96.6%	78.2%	0.6223	0.9841	0.355
RLE-CUI-HUI-LSI	0.969(0.935-1.000)	0.442	93.1%	93.6%	0.844	0.973	0.514
ALBI	0.639(0.515–0.763)	-2.742	55.2%	71.8%	0.4212	0.8117	0.457
MELD	0.634(0.522–0.745)	3.964	79.3%	50.0%	0.3709	0.8666	0.070
FLR	0.644(0.515–0.773)	714.270	44.8%	85.9%	0.5416	0.8072	0.457

^*^ Referred to *p*<0.05; *AUC*, area under the curve; *CI*, confidence interval; *H-L*, hosmer–lemeshow; *PPV*, positive predictive value; *NPV*, negative predictive value; *RLE*, relative liver enhancement; *CUI*, contrast uptake index; *HUI*, hepatic uptake index; *LSI*, liver-to-spleen index; *TBil*, total bilirubin; *FLR*, future liver remnant; *ALBI*, albumin-bilirubin; *MELD*, model for end-stage liver disease.

The H-L test results indicated that, except for the CUI model, all other models had good goodness of fit (all *p* > 0.05), suggesting high consistency between the model-predicted probabilities and the actual performance observed probabilities.

Pairwise comparisons of the AUCs among all models were performed using DeLong’s test. The results showed that the predictive performance of the RLE-TBil model was significantly superior to that of the HUI model, LSI model, TBil model, and all conventional liver function indices. (**Supplementary Table 1**).

Internal validation was performed using 1000 bootstrap resamples. The results revealed minimal optimism bias (absolute value< 0.02) for all models, and the corrected AUC values were highly consistent with the original AUC values, indicating favorable stability and no significant overfitting (**Supplementary Table 2**).

**Figure 4:**
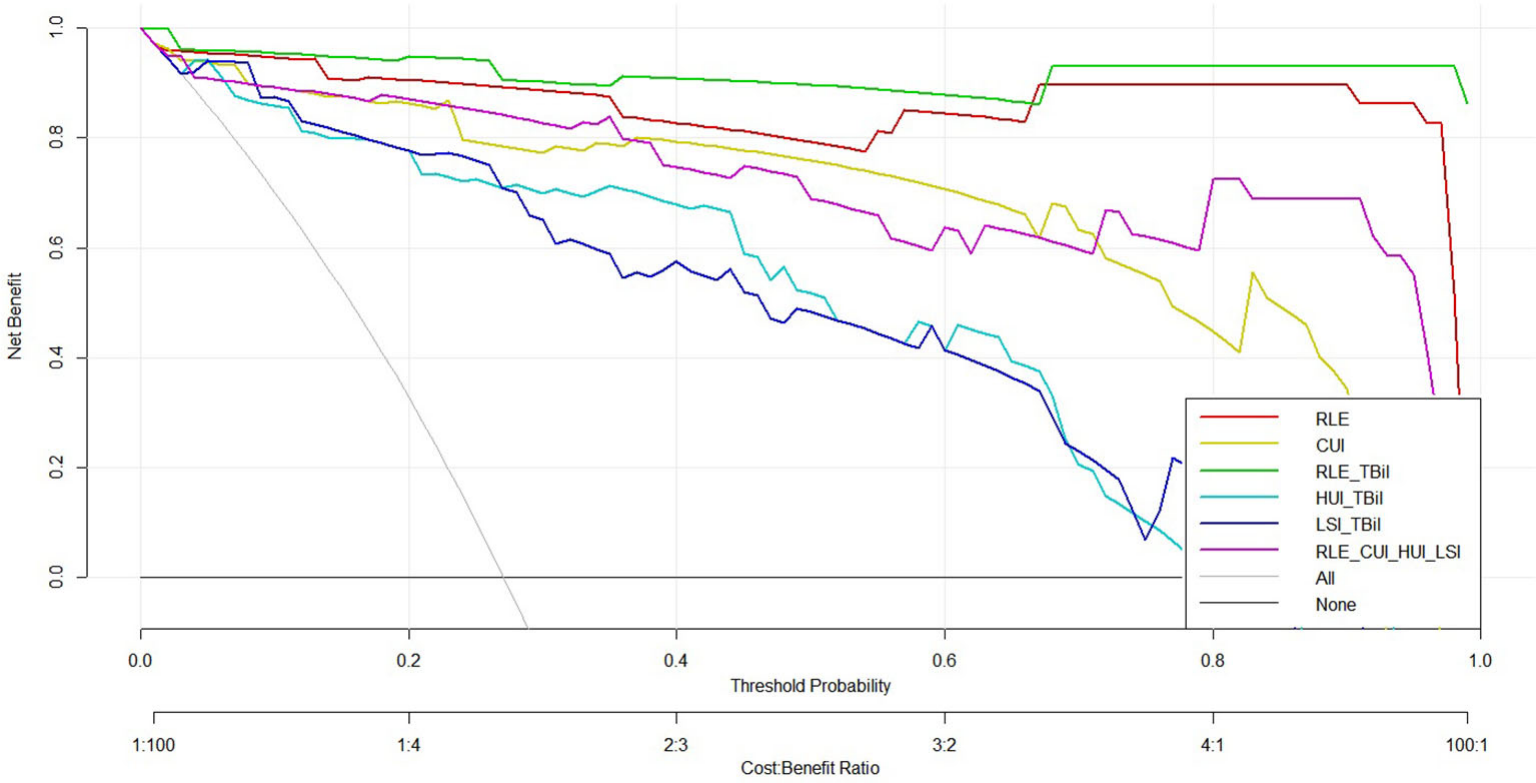
DCA of six optimal prediction models for PHLF. The y-axis represents the standardized net benefit, and the x-axis indicates the high-risk threshold probability. The six optimal models included RLE, CUI, RLE-TBil, HUI-TBil, LSI-TBil, and RLE-CUI-HUI-LSI. All models yielded favorable clinical net benefits, among which the RLE-TBil combined model presented the best predictive performance and superior clinical benefit compared with the other models.

DCA was performed to further evaluate the clinical utility of these models. The DCA results for the six optimal prediction models are shown in **Figure 4**. Within the clinically relevant range of threshold probabilities, all models based on quantitative imaging biomarkers of liver function achieved a significantly higher net benefit than the strategies of treating all or none of the patients. Among them, the RLE-TBil model demonstrated the highest net benefits, which were markedly superior to those of conventional liver function indices, indicating favorable clinical utility for decision-making. The full DCA curves for all 12 models are shown in **Supplementary Figure 1**.

### Stratified analysis of the RLE-TBil model by the extent of hepatectomy

To confirm the generalizability of the RLE-TBil model across different surgical contexts, we performed a stratified analysis according to hepatectomy extent. In the hemihepatectomy subgroup (n = 94), the model maintained excellent predictive performance, with an AUC of 0.998 (95% CI: 0.994-1.000, *p* < 0.001). Logistic regression confirmed that RLE remained an independent protective factor (*p* = 0.023), whereas TBil showed a positive correlation with PHLF risk (*p* = 0.054), which is consistent with the results from the overall cohort. Due to the small sample size (n = 13) and limited number of PHLF events (n = 3) in the extended hemihepatectomy subgroup, stable model fitting could not be achieved; thus, the results from this subgroup were not reported.

### Calibration and precision-recall performance of the RLE-TBil model

**Figure 5:**
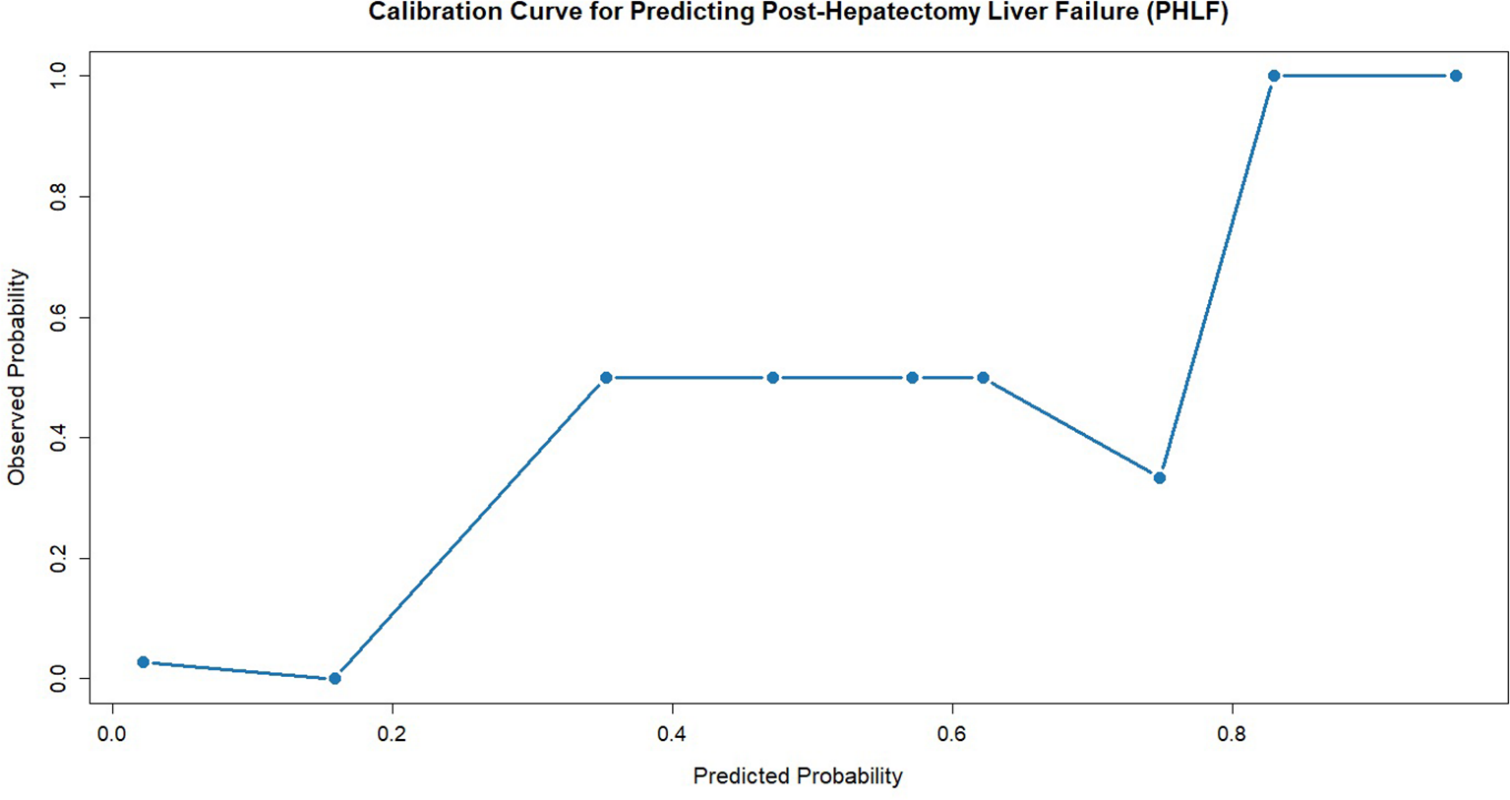
Calibration curve of the RLE-TBil prediction model. The curve illustrates the agreement between model-predicted probabilities and observed PHLF outcomes. The diagonal line represents perfect calibration.

The calibration curve demonstrated good agreement between the predicted probabilities and observed PHLF outcomes (**Figure 5**). The points were distributed close to the ideal diagonal line across the full range of predicted probabilities, indicating reliable calibration of the model. The Hosmer-Lemeshow test further confirmed that there was no significant deviation from perfect calibration (*p* > 0.05).

**Figure 6:**
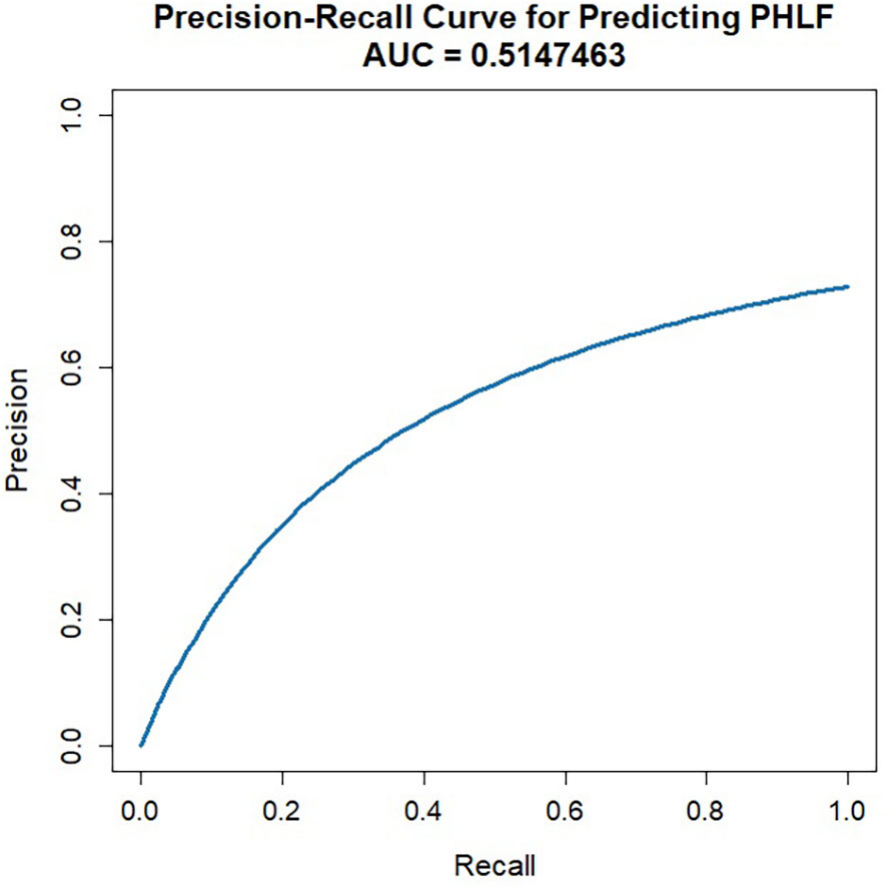
Precision-recall curve of the RLE-TBil prediction model. The curve reflects the trade-off between precision and recall in predicting PHLF, with an area under the curve (AUC) of 0.515.

The precision-recall curve (**Figure 6**) yielded an AUC of 0.515. This moderate performance reflects the trade-off between precision and recalls in the imbalanced dataset (29 PHLF vs. 78 non-PHLF cases), where the model prioritizes high recall (sensitivity) to correctly identify all of the PHLF cases at the cost of a small number of false-positive predictions.

To further validate the clinical interpretability of the RLE-TBil model, we performed a misclassification analysis at an optimal Youden index cutoff of 0.086. The model achieved a true-positive rate of 100% and correctly identified all 29 PHLF cases. These true-positive patients exhibited significantly higher mean preoperative RLE (3.872 vs. 2.613) and TBil (32.14 vs. 15.474) levels compared with the overall cohort, consistent with the pathophysiological basis of PHLF. Conversely, only four false-positive cases were identified (false-positive rate: 3.7%). These patients had lower mean RLE values (1.601 vs. 2.613) but mildly elevated TBil levels (19.025 vs. 15.474), with all patients presenting with ALBI grades 1–2 and no severe baseline liver dysfunction. This pattern indicates that the model predictions are biologically plausible rather than random noise, reducing concerns about overfitting.

## Discussion

This study has established several preoperative prediction models for PHLF based on quantitative imaging biomarkers of liver function derived from gadoxetic acid-enhanced MRI. The results demonstrated that all models based on quantitative imaging biomarkers exhibited favorable predictive performance for PHLF, and their performance was significantly superior to that of conventional liver function indices such as TBil, ALBI score, MELD score, and FLR. Among them, the RLE-TBil combined model presented the best predictive efficacy, followed by the RLE model; the RLE-CUI-HUI-LSI model also achieved a strong diagnostic performance.

In this study, the baseline data of the enrolled patients were compared between groups using the independent-samples t-test or Mann-Whitney U test, and indices with statistically significant intergroup differences were initially screened. Previous studies have indicated that during the HBP, gadoxetic acid is taken up by functional hepatocytes via organic anion-transporting polypeptides (OATP B1/B3) and excreted via the renal and biliary pathways through multidrug resistance-associated proteins (MRP2/3). This process is independent of hepatic blood perfusion. Consequently, the degree of liver enhancement can directly reflect the quality and functional status of viable hepatocytes (17–19). The baseline data in this study demonstrated that RLE, CUI, HUI, and LSI were significantly lower in the PHLF group than in the non-PHLF group (*p* < 0.05) (**Table 1**), indicating impaired hepatocellular uptake and reduced total functional hepatocytes in patients with PHLF. These findings are consistent with those of previous studies and further support the reliability of quantitative MRI parameters for evaluating hepatic functional reserve.

Consistent with the study by Morandi et al. (8), significant differences in ALBI score and MELD score were observed between the two groups in this study, verifying the clinical value of conventional liver function evaluation systems. The extent of hepatectomy, widely recognized as an important factor affecting PHLF (24), was also included in the analysis in this study. Although the difference was not statistically significant, a potential impact trend was suggested. However, there was no significant difference between the two groups, which was inconsistent with the results of previous studies. A possible reason for this is that all patients in this study underwent major hepatectomy with rigorous preoperative liver function evaluation and surgical planning, maintaining the FLR within a relatively safe range and thus weakening its independent predictive effect. In the future, this will be further verified by expanding the sample size and including patients with different surgical resection ranges (3).

Subsequently, the above indices with statistically significant differences were included in the univariate and multivariate regression analyses to further identify key factors with independent predictive value for PHLF. Based on the results of multivariate regression analysis, multiple preoperative predictive models for PHLF were developed in this study, including single-index models (RLE, CUI, HUI, and LSI), combined models of quantitative imaging biomarkers with TBil (RLE-TBil, HUI-TBil, and LSI-TBil), and a comprehensive model constructed by integrating multiple parameters via factor analysis (RLE-CUI-HUI-LSI). Notably, TBil showed an independent predictive value in the RLE-TBil model, but failed to remain significant in the RLE-CUI-HUI-LSI comprehensive model, mainly because the composite imaging index already captures most of the prognostic information related to liver functional reserve, thus reducing the additional predictive contribution of TBil.

Furthermore, ROC curve analysis was comprehensively performed to compare the predictive performance of each model with that of the conventional liver function indices (ALBI score, MELD score, and FLR). The results demonstrated that all models constructed based on gadoxetic acid-enhanced MRI quantitative liver function imaging biomarkers demonstrated robust predictive performance for PHLF and were found to be significantly superior to conventional liver function evaluation indices. Among the single-index models, RLE achieved the optimal predictive performance. Some combined models showed improved predictive trends compared to individual imaging biomarkers. Ultimately, the RLE-TBil combined model yielded the highest AUC, optimal sensitivity, and specificity, and was thus identified as the best predictive model in this study.

To interpret the predictive performance of our RLE-TBil model better, we compared it with two recent representative studies that focused on preoperative PHLF evaluation based on gadoxetic acid-enhanced MRI.

Jeong et al. published a multicenter study in European Radiology (2024) (27), constructing deep learning-assisted predictive models in 1760 patients and reported an optimism-corrected AUC of 0.81. The high predictive efficiency observed in our cohort (AUC 0.997) can be explained by several factors. Our combined model integrates hepatocellular uptake indicator and serum excretory markers, enabling a more comprehensive reflection of liver functional changes. Strictly unified inclusion and exclusion criteria were applied in our single-center cohort to reduce clinical heterogeneity, and multiple statistical validations were performed to ensure model stability and minimize overfitting risk. Compared with automatic deep learning segmentation, the manually defined ROI in our work is more convenient for routine clinical applications, although we recognize that its cross-center reproducibility is relatively limited.

Another relevant study from Bartholomä et al. (British Journal of Surgery, 2025) (12) established the sFLR-HUI scoring system in international multicenter cohorts, achieving an AUC of 0.803 when combined with MELD3. Their scheme relies on highly standardized imaging protocols and segmentation procedures, which favors unified multicenter research. In contrast, our RLE-TBil model adopts readily available clinical and imaging indicators and is more suitable for rapid individualized risk assessment in daily single-center practice. Nevertheless, we fully acknowledge that the external generalizability of our current model remains insufficient.

Furthermore, unlike the nomogram and online calculator used in Jeong et al. ‘s study, we did not develop similar auxiliary tools at this stage. This was mainly attributed to the retrospective single-center design. We will therefore consider constructing predictive nomograms and convenient calculation tools after future prospective multicenter validations to further promote the clinical transformation of our model.

In addition to its excellent discriminative ability, the RLE-TBil model showed good calibration, with a calibration curve demonstrating favorable consistency between the predicted probabilities and actual observed outcomes. The Hosmer-Lemeshow test confirmed no significant deviation from perfect calibration, indicating that the model’s risk estimates were reliable and clinically interpretable. While the ROC-AUC of 0.997 indicated excellent discriminative ability, the PR-AUC of 0.515 reflected the inherent challenge of predicting a relatively uncommon event with limited positive cases. This trade-off between high sensitivity and moderate precision should be considered when applying this model to clinical decision-making. Specifically, the model achieved 100% sensitivity at the cost of limited precision (PPV = 87.94%), which means that it rarely misses true PHLF cases, but may lead to unnecessary conservative management in a small subset of patients. The relatively modest performance of the precision-recall curve was mainly attributed to the imbalance between positive and negative cases in our cohort, reflecting a necessary clinical trade-off to prioritize high sensitivity in identifying true PHLF patients. Further misclassification analysis of true and false-positive cases verified that the predictive pattern was biologically reasonable rather than caused by random overfitting, which further supported the robustness and clinical applicability of this model.

Based on the pathophysiological basis (28), the outstanding predictive advantage of the RLE-TBil model is biologically plausible. RLE can accurately reflect the uptake capacity of hepatocytes for gadoxetic acid, whereas TBil mainly reflects the biliary excretion function of the liver. These two biomarkers correspond to the key links of hepatic “uptake-excretion” and complement each other. Their combined use enables a more comprehensive evaluation of the overall functional status of hepatocytes and compensates for the limitation that single biomarkers only reflect partial liver function, thus achieving higher predictive performance than single imaging biomarkers or conventional biochemical markers.

We performed a stratified analysis according to the extent of hepatectomy to further validate the robustness of the model across different surgical contexts. The results showed that the RLE-TBil model maintained excellent predictive performance in the hemihepatectomy subgroup, with consistent effect directions for key variables compared to the overall cohort. However, owing to the small sample size and limited number of PHLF events in the extended hemihepatectomy subgroup, stable model fitting could not be achieved. This therefore suggests that, while the model is highly reliable for patients undergoing conventional hemihepatectomy, its generalizability to extended hepatectomy cases requires validation in larger cohorts.

As a supplementary analysis to the univariate models, additional diagnostic checks were performed for the CUI-based indices included in the models. Cook’s distance and studentized residuals identified one influential outlier that caused suboptimal calibration of the univariate CUI model. Excluding this case, good calibration was restored (H-L *p* = 0.389), and the Box-Tidwell test with a quadratic term sensitivity analysis confirmed the robustness of the CUI’s predictive effect.

Several comprehensive models established in this study integrate quantitative liver function parameters derived from gadoxetic acid-enhanced MRI and biochemical indices. These models can effectively identify preoperative high-risk populations for PHLF among patients with HCC who underwent major hepatectomy. Derived solely from the HBP sequence, these models are simpler than T1 mapping and elastography and they are more suitable for routine clinical applications (29). Featuring objectivity, noninvasiveness, and easy accessibility, they provide reliable evidence for preoperative risk stratification, surgical planning, and individualized perioperative management, thereby ensuring surgical safety and improving perioperative prognosis.

## Limitations

This study has several limitations. First, as a single-center retrospective study, patients were exclusively enrolled from West China Hospital, Sichuan University, resulting in a relatively homogeneous study cohort. A total of 55 patients were excluded because of the absence of standardized liver function and biochemical indices within 5 days after surgery. Such nonrandom exclusion may introduce selection bias, leading to systematic differences in clinical characteristics between excluded and included patients. To address this, we will compare the baseline characteristics between these two groups to quantify potential bias, standardize the timing of postoperative liver function assessments to reduce data loss, and further perform multicenter external validation with a temporal validation cohort of subsequent patients to correct for bias and enhance the generalizability of our findings. In addition, all of the imaging analyses were performed using manually defined ROIs, which, while accessible for routine clinical use, may introduce inter-reader variability and lower cross-center reproducibility than automated segmentation workflows. Future work will focus on establishing standardized ROI delineation protocols to improve measurement consistency. Second, the enrolled patients were confined to those with chronic hepatitis B-related HCC, and the influence of different underlying liver disease etiologies on the model performance was not adequately explored. Underlying liver disease is a key determinant of liver regeneration capacity and is closely associated with PHLF (30). Unlike most Western countries, where hepatitis C virus infection and non-alcoholic fatty liver disease are the main causes of HCC, most patients with HCC in China have a history of chronic hepatitis B virus infection with liver function impairment (31). Future studies are needed to validate the generalizability of the present model to patients with HCC of different etiologies. Third, all the included patients underwent major hepatectomy, including hemihepatectomy or extended hemihepatectomy. Given the uniform background of extensive hepatic resection, there was no significant difference in the FLR between the groups, limiting the predictive value of the FLR for PHLF (*p* = 0.166), which is inconsistent with previous studies. This indicates that the predictive efficacy of the FLR is influenced by the extent of liver resection. In addition, due to the small sample size of the extended hepatectomy subgroup, stable and reliable subgroup analysis results could not be obtained. Future studies with expanded sample sizes and inclusion of patients undergoing different resection extents are needed in order to further elucidate the relationship between surgical scope and PHLF and to validate the predictive stability of FLR and imaging biomarkers. Fourth, stratified analysis of PHLF severity was not performed in this study. Given that predictive models and clinical management strategies vary significantly across different grades of PHLF, further stratified studies are warranted to clarify the performance of these quantitative liver function biomarkers in predicting PHLF of different severities. Fifth, although the RLE-TBil model has demonstrated excellent discriminative performance in this cohort, the potential risk of overfitting cannot be entirely excluded. The single-center retrospective design, relatively limited sample size, and high exclusion rate (40%) may have contributed to an overly optimistic estimation of model performance by reducing cohort heterogeneity. Even after internal verification using the bootstrap method, external validation based on independent multi-center prospective cohorts is essential to confirm the robustness and generalizability of the model in future clinical applications. Notably, the current lack of dedicated clinical application tools, such as nomograms or open-access online calculators, further limits the immediate translation of our findings into routine practice, which will be addressed in subsequent multi-center validation studies.

## Conclusion

In conclusion, the predictive models constructed in this study based on gadoxetic acid-enhanced MRI quantitative imaging biomarkers of liver function demonstrate high accuracy in predicting PHLF and can provide objective evidence for clinical preoperative risk stratification, optimization of surgical planning, and formulation of individualized perioperative management strategies.

## Data Availability

The de-identified research dataset of this study is available from the corresponding author upon reasonable request. Due to medical ethics and patient privacy protection policies, the original data cannot be directly uploaded to public databases. Qualified researchers may obtain the de-identified research data after submitting a reasonable request and obtaining approval from the Ethics Committee of West China Hospital, Sichuan University. Requests to access the datasets should be directed to Yuan Yuan, yuanyuan1686@126.com.

## Supplementary material

Supplementary Figure 1 DCA of all 12 prediction models for PHLF. The y-axis represents the standardized net benefit, and the x-axis indicates the high-risk threshold probability. This supplementary figure provides the complete DCA curves for all evaluated models.

Supplementary Table 1 DeLong test *p-*value matrix for all models ^*^ referred to *p*<0.05; DeLong test was used for comparison, *RLE* relative liver enhancement, *CUI* contrast uptake index, *HUI* hepatic uptake index, *LSI* liver-to-spleen index, *TBil* total bilirubin, *FLR* future liver remnant, *ALBI* albumin-bilirubin, *MELD* model for end-stage liver disease.

Supplementary Table 2 bootstrap internal validation results of all predictive models. *Internal validation was performed using 1000 bootstrap resamples to assess model robustness and overfitting risk. *AUC* area under the curve, *RLE* relative liver enhancement, *CUI* contrast uptake index, *HUI* hepatic uptake index, *LSI* liver-to-spleen index, *TBil* total bilirubin,*FLR* future liver remnant, *ALBI* albumin-bilirubin, *MELD* model for end-stage liver disease.
