# Supplementary figures and images for: Preoperative evaluation of post-hepatectomy liver failure in hepatocellular carcinoma based on gadoxetic acid-enhanced MRI
Source: Front Oncol. 2026 Jun 29;16:1789478. doi: 10.3389/fonc.2026.1789478 (PMC13357168; doi:10.3389/fonc.2026.1789478)

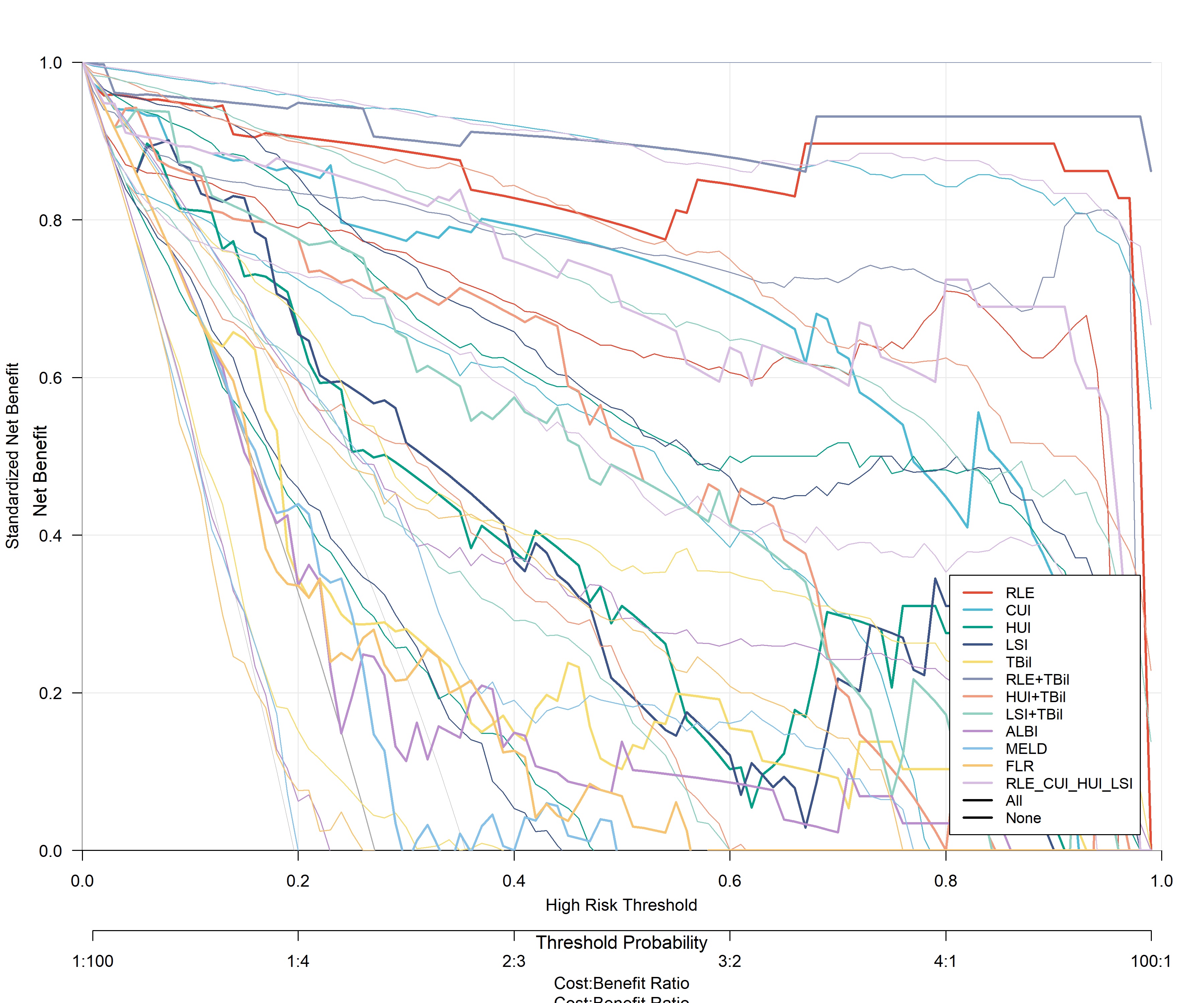

Supplement: Supplementary Figure 1 — DCA of all 12 prediction models for PHLF. The y-axis represents the standardized net benefit, and the x-axis indicates the high-risk threshold probability. This supplementary figure provides the complete DCA curves for all evaluated models. [file Image1.jpg]
