# Supplementary material for: Preoperative evaluation of post-hepatectomy liver failure in hepatocellular carcinoma based on gadoxetic acid-enhanced MRI
Source: Front Oncol. 2026 Jun 29;16:1789478. doi: 10.3389/fonc.2026.1789478 (PMC13357168; doi:10.3389/fonc.2026.1789478)
Supplement: Supplementary Table 1 — DeLong test p-value matrix for all models * referred to p<0.05; DeLong test was used for comparison, RLE relative liver enhancement, CUI contrast uptake index, HUI hepatic uptake index, LSI liver-to-spleen index, TBil total bilirubin, FLR future liver remnant, ALBI albumin-bilirubin, MELD model for end-stage liver disease. [file Table1.docx]

Supplementary Tables

- **Supplementary Table 1** DeLong test *p-*value matrix for all models

| Model | RLE | CUI | HUI | LSI | TBil(μ mol/L) | RLE-TBil | HUI-TBi | LSI-TBil | RLE-CUI-HUI-LSI | ALBI | MELD | FLR |
| --- | --- | --- | --- | --- | --- | --- | --- | --- | --- | --- | --- | --- |
| RLE | 1.0000 | 0.0798 | 0.0003 | 0.0014 | <0.0001 | 0.2777 | 0.0400 | 0.0309 | 0.0881 | <0.0001 | <0.0001 | <0.0001 |
| CUI | 0.0798 | 1.0000 | 0.0074 | 0.0347 | 0.0003 | 0.0828 | 0.4247 | 0.3697 | 0.3951 | <0.0001 | <0.0001 | <0.0001 |
| HUI | 0.0003 | 0.0074 | 1.0000 | 0.0773 | 0.0419 | 0.0003 | 0.0209 | 0.0372 | 0.0001 | 0.0022 | 0.0007 | 0.0015 |
| LSI | 0.0014 | 0.0347 | 0.0773 | 1.0000 | 0.0122 | 0.0005 | 0.0877 | 0.0938 | 0.0010 | 0.0005 | 0.0001 | 0.0006 |
| TBil(μ mol/L) | <0.0001 | 0.0003 | 0.0419 | 0.0122 | 1.0000 | <0.0001 | 0.0001 | 0.0001 | 0.0001 | 0.1245 | 0.0288 | 0.3357 |
| RLE-TBil | 0.2777 | 0.0828 | 0.0003 | 0.0005 | <0.0001 | 1.0000 | 0.0074 | 0.0041 | 0.0864 | <0.0001 | <0.0001 | <0.0001 |
| HUI-TBil | 0.0400 | 0.4247 | 0.0209 | 0.0877 | 0.0001 | 0.0074 | 1.0000 | 0.7237 | 0.1123 | <0.0001 | <0.0001 | <0.0001 |
| LSI-TBil | 0.0309 | 0.3697 | 0.0372 | 0.0938 | 0.0001 | 0.0041 | 0.7237 | 1.0000 | 0.0952 | <0.0001 | <0.0001 | <0.0001 |
| RLE-CUI-HUI-LSI | 0.0881 | 0.3951 | 0.0001 | 0.0010 | 0.0001 | 0.0864 | 0.1123 | 0.0952 | 1.0000 | <0.0001 | <0.0001 | <0.0001 |
| ALBI | <0.0001 | <0.0001 | 0.0022 | 0.0005 | 0.1245 | <0.0001 | <0.0001 | <0.0001 | <0.0001 | 1.0000 | 0.9435 | 0.9565 |
| MELD | <0.0001 | <0.0001 | 0.0007 | 0.0001 | 0.0288 | <0.0001 | <0.0001 | <0.0001 | <0.0001 | 0.9435 | 1.0000 | 0.9093 |
| FLR | <0.0001 | <0.0001 | 0.0015 | 0.0006 | 0.3357 | <0.0001 | <0.0001 | <0.0001 | <0.0001 | 0.9565 | 0.9093 | 1.0000 |

^*^ referred to *p*<0.05; DeLong test was used for comparison, *RLE* relative liver enhancement, *CUI* contrast uptake index, *HUI* hepatic uptake index, *LSI* liver-to-spleen index, *TBil* total bilirubin, *FLR* future liver remnant, *ALBI* albumin-bilirubin, *MELD* model for end-stage liver disease.

- **Supplementary Table 2** bootstrap internal validation results of all predictive models

| Model | Original AUC | Optimism Bias | bootstrap-corrected AUC |
| --- | --- | --- | --- |
| RLE | 0.985 | <0.001 | 0.985 |
| CUI | 0.957 | 0.001 | 0.957 |
| HUI | 0.873 | <0.001 | 0.873 |
| LSI | 0.895 | 0.001 | 0.894 |
| TBil(μ mol/L) | 0.733 | -0.002 | 0.735 |
| RLE-TBil | 0.997 | <0.001 | 0.997 |
| HUI-TBil | 0.933 | <0.001 | 0.933 |
| LSI-TBil | 0.931 | -0.001 | 0.931 |
| RLE-CUI-HUI-LSI | 0.969 | -0.001 | 0.969 |
| ALBI | 0.639 | -0.014 | 0.653 |
| MELD | 0.634 | -0.004 | 0.638 |
| FLR | 0.644 | <0.001 | 0.644 |

^*^Internal validation was performed using 1000 bootstrap resamples to assess model robustness and overfitting risk. *AUC* area under the curve, *RLE* relative liver enhancement, *CUI* contrast uptake index, *HUI* hepatic uptake index, *LSI* liver-to-spleen index, *TBil* total bilirubin,*FLR* future liver remnant, *ALBI* albumin-bilirubin, *MELD* model for end-stage liver disease.
